# Impact of molar teeth distalization by clear aligners on maxillary alveolar bone thickness and root resorption: a three‑dimensional study

**DOI:** 10.1186/s12903-024-03987-3

**Published:** 2024-02-14

**Authors:** Naseem Ali Al-Worafi, Bowen Zheng, Leena Ali Al-Warafi, Enas Senan Alyafrusee, Majedh Abdo Ali Alsomairi, Yi Liu

**Affiliations:** 1 Department of Orthodontics, School and Hospital of Stomatology, China Medical University, Clinical Medical Research Center of Orthodontic Disease, Shenyang 110002, P.R. China; 2 Department of Orthodontics, Faculty of Dentistry, First Affiliated Hospital of Zhengzhou University, Zhengzhou, P.R. China

**Keywords:** Alveolar bone thickness, Clear aligners, CBCT, Maxillary molars, Molar distalization, Root resorption

## Abstract

**Objective:**

This study aimed to evaluate the impact of molar teeth distalization movement by clear aligners on changes in the alveolar bone thickness and orthodontically induced inflammatory root resorption (OIIRR) in maxillary molars using conebeam computed tomography (CBCT).

**Materials and methods:**

Three-dimensional CBCT scans of 35 adult patients (one hundred forty maxillary molars) with pre-designed selection criteria and a mean age of 24.4 ± 7.1 years were included. The measured parameters, including alveolar bone thickness for maxillary molars and root resorption (OIIRR), were analyzed using pre-and post-treatment CBCT (T0 and T1, respectively) with Invivo 6.0 software.

**Result:**

Post-treatment, relevant statistically significant changes included deposition of bone in the average palatal surface of the 1st molars. The reduction of bone was seen in the average buccal surface of the first molars and both surfaces of the second molars. Regarding root length after treatment, the average maxillary 1st molar roots showed significant OIIRR (*p* < 0.001).

**Conclusion:**

Clear aligner treatment could effectively reduce the incidence of alveolar bone thickness reduction and OIIRR in treating Class II malocclusions compared to conventional braces, as shown in previous studies. This research will aid in fully grasping the benefits of clear aligners.

## Introduction

Skeletal Class II malocclusions, considered the most common in orthodontic patients, can affect the patient’s facial appearance, masticatory function, and mental health [1]. During the correction of malocclusion, orthodontic force may influence the alveolar bone remodeling. Therefore, the anatomy of the alveolar bone plays a crucial role in orthodontic treatment, as it dictates the treatment’s direction through coordination of resorption and apposition [2]. When there is a significant conflict between the bone remodeling and the amount of tooth movement occurring at a 1:1 ratio, the tooth remains within the alveolar housing “with the bone” [3]. Molar distalization is one of the most critical orthodontic movements for correcting skeletal Class II malocclusions [4, 5]. This technique can move the teeth away from the alveolar bone housing “through-the-bone” [3], potentially causing root resorption, alveolar bone loss, dehiscence, fenestration, and gingival recession [6]. 

Fixed appliances have been mainstreamed in orthodontic treatment [7]. However, patient acceptance is often hindered by challenges in maintaining proper oral health and the aesthetic concerns associated with the appliances [8]. Clear aligners, such as the Invisalign system, have become increasingly popular due to their advantages in comfort and aesthetics compared to conventional fixed appliances [9, 10]. Sequential distalization movement with clear aligners may be programmed in the maxillary arch for correction of a Class II relationship, with high predictability of molar distalization (88%) when supported by attachments on the tooth surface [11, 12] with or without posterior interproximal reduction and/or elastic simulation [13]. In addition, it is crucial to include bone thickness, dehiscence, fenestration, and other intra-bony defects in the orthodontic diagnosis and treatment plans [14, 15]. The accuracy of measurements may also be influenced by the tools used for assessment [7]. Prior research has primarily focused on assessing alterations in the alveolar bone and identifying orthodontically induced inflammatory root resorption (OIIRR) before and after orthodontic treatment, typically using periapical or bitewing radiographs [16, 17]. However, the reliability of these findings is constrained by the limitations of two-dimensional images, such as magnification, geometric distortion, and structural overlap [18].

To the authors’ knowledge, there are few published studies on using CBCT to assess changes in the alveolar bone thickness and root length in anterior teeth after distalization movements with conventional fixed orthodontic appliances [19]. Moreover, there is a lack of data evaluating three-dimensional changes in alveolar bone and OIIRR following maxillary molar distalization movement with clear aligners. Therefore, this study aims to assess the changes in alveolar bone thickness and OIIRR three-dimensionally following maxillary molar distalization with clear aligners in patients with Class II malocclusions using CBCT.

## Materials and methods

### Patients’ selection

This retrospective study was conducted with the approval of the Ethics Committee of China Medical University Stomatological School and adhered to the principles of the Declaration of Helsinki. The Research Ethics Committee endorsed the informed consent process, and participants provided written informed consent. The study analyzed CBCT images of 35 adult patients (mean age of 24.4 ± 7.1 years) who were receiving clear aligner therapy (Invisalign, Align Technology, San Josè, California, USA) and were treated with sequential maxillary arch distalization as suggested by Align Technology and described by Ravera [20].

The treatment involved using Class II elastics and rectangular vertical attachments on the maxillary molars and premolars. Each case was meticulously planned to achieve sequential distalization of the upper arch, with the staging set at 0.25 mm per aligner. The distalization began with the maxillary second molars; once they moved two-thirds of the desired distance, the first molars, followed by the premolars, and so on, moved back sequentially until the en masse retraction of the four incisors completed the treatment plan. The protocol included using attachments and Class II elastics to retract the premolars, canines, and incisors. As the molars were distalized using the aligners, they acted as an anchor against the rest of the arch. To prevent loss of anchorage and potential flaring of the anterior teeth, Class II elastics (1/4-inch, 4.5 oz from Ormco Corp., Glendora, CA, USA) were employed to reinforce anchorage.

### Selection criteria

All patients treated with clear aligners who were screened between 2017 and 2022 were evaluated using CBCT before (T0) and after (T1) treatment. The inclusion criteria included: (1) participants aged between 17 and 32 years; (2) classified skeletal Class II with malocclusion of > 4.7^o^ according to the ANB angle norms specific to their ethnic group [21]; (3) having adequate space for molar distalization without the need for temporary anchorage devices; (4) eruption of all permanent teeth except the third molar; (5) complete root formation; and (6) mild to moderate crowded dental arches. Exclusion criteria were: (1) patients with root caries or fractures; (2) periodontal or gingival issues at the onset of treatment; (3) extraction treatment except for third molars; (4) History of craniofacial syndromes or bone diseases; (5) low-quality CBCT scans at T0 and/or T1; (6) evidence of prior inflammatory root resorption; and (7) prior early interceptive or comprehensive orthodontic treatment.

The sample size for this study was calculated using G*Power software (v3.1.3; Franz Faul, Universität Kiel, Germany), drawing from a previous study [7] that depended on the palatal bone thickness of the maxillary central incisor. The calculation aimed for a 95% power level with a significance level of 5% (α = 0.05) and an effect size (dz = 0.9), based on mean values of 1.51 ± 0.52 and 1.24 ± 0.7 for pre- and post-treatment, respectively. This analysis determined that a minimum of 18 subjects would be necessary. Ultimately, our study included 35 subjects, exceeding the calculated requirement.

### CBCT analysis

The analysis involved the examination of CBCT images acquired from the I-CAT Imaging System (KaVo Company, Germany). These images were obtained using established protocols and were administered by a specialized radiographer. The settings for image acquisition were configured as follows: 120 kV, 5 mA, a field of view measuring 23 cm × 17 cm, an exposure time of 17.8 s, a voxel size of 0.3 mm, and a slice thickness of 2 mm. The images were uniformly repositioned to align with the Frankfort-horizontal (FH) plane in the sagittal plane. Subsequently, CBCT scans collected at T0 and T1 were converted into DICOM (Digital Imaging and Communication in Medicine) file format and subsequently imported into the Invivo 6.0 software (Anatomage, San Jose, CA, USA). Within this software, the section view features, which enable three-dimensional visualization of X, Y, and Z sections (representing axial, coronal, and sagittal planes, respectively), were utilized for analysis.

**Fig. 1:**
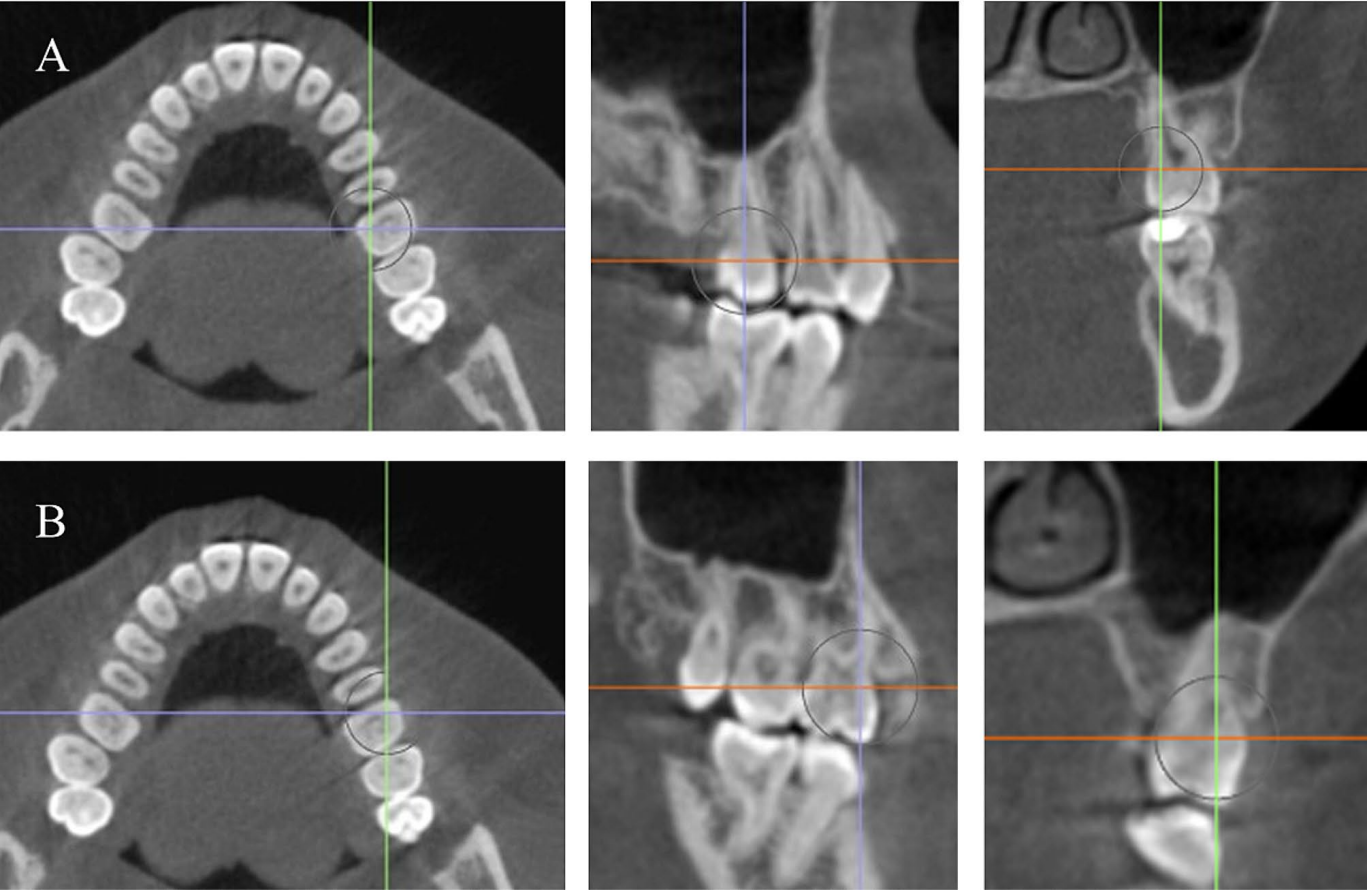
(**A**) Orientation of the axial, sagittal, and coronal planes of the palatal root to be perpendicular to the long axis of each tooth under assessment. (**B**) Orientation of the axial, sagittal, and coronal planes of the mesiobuccal root to be perpendicular to the long axis of each tooth under assessment

**Fig. 2:**
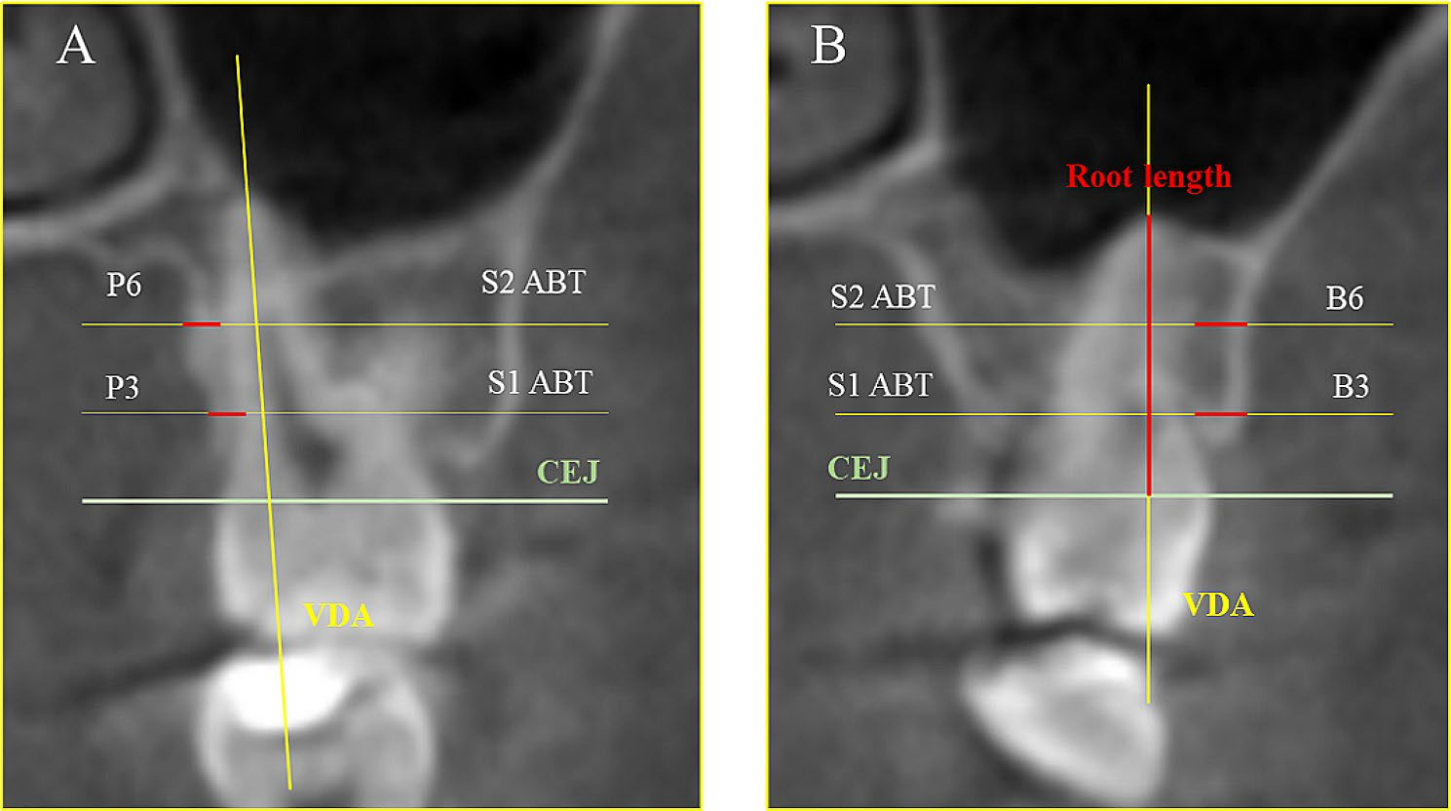
In the coronal view, the reference lines are represented by VDB: vertical dental axis (yellow line) and CEJ: cementoenamel junction (green line). The measurements represented by ABT are the alveolar bone thickness at S1 and S2 (**A**) P3 and P6 represent the palatal side 3- and 6-mm distance from the CEJ in the apical direction. (**B**) B3 and B6 represent the buccal side 3- and 6-mm distance from the CEJ in the apical direction (red color) and root length: the distance between the constructed CEJ line and the root?s most apical point (red line)

The sagittal, axial, and coronal views were properly oriented following the methodology of Ma et al. [1] as shown in Fig. 1. To measure the thickness of the buccal surface of the maxillary molars, the vertical dental axis (VDA) was drawn from the tip of mesiobuccal cusp to the apex of the mesiobuccal root (Fig. 2, A). Similarly, for the palatal surface, the VDA was drawn from the tip of the palatal cusp to the apex of the palatal root. The buccal and palatal bone thicknesses were measured at two sites: the distance from the buccal/palatal cortex to the tooth root surface at the level 3 mm (S1) below the cementoenamel junction (CEJ) and the distance at the mid-root level 6 mm (S2) below the CEJ (as indicated in Fig. 2, B).

The four measurements were taken for each tooth (two buccal and two palatal) before treatment (T0). These measurements were repeated after treatment completion (T1). For OIIRR measurements, only the mesiobuccal tooth of the molar teeth was chosen. The roots length was represented by the distance between the CEJ and apex (as shown in Fig. 2, A).

### Statistical analysis

The statistical analysis was calculated utilizing IBM SPSS Statistics V. 26.0 software (IBM Corp.). Descriptive statistics were calculated and presented, including each variable’s mean and standard deviation. The normality data was evaluated using Shapiro-Wilk’s test. A paired t-test was performed for comparisons between pre- and post-treatment measurements. The reproducibility and reliability of measurements for alveolar bone thickness and OIIRR were assessed using the intraclass correlation coefficient (ICC) along with absolute and relative technical measurement errors (TEM and rTEM). The statistically significant level was set as *P* < 0.05.

## Results

A total of one hundred forty molar teeth roots from 35 patients who met the inclusion criteria were analyzed, with a mean age of 24.4 ± 7.1 years. The reliability of intra- and inter-observer assessments for repeated measurements showed non-significant differences, where both intra- and interobserver R and ICC values were excellent to very good (see Table 1). The positive values indicated bone losses and root resorption, while the negative values denoted bone deposition in the horizontal plane.

**Table 1:** Reliability analysis of all measurements used in this study

Variable	Intra-observer reliability				Inter-observer reliability			
	ICC	TEM	RTEM	R	ICC	TEM	RTEM	R
U6BS1	0.980	0.00	0.42	0.999	0.961	1.11	7.85	0.975
U6BS2	0.941	0.99	6.99	0.979	0.824	0.94	6.64	0.986
U6PS1	0.845	0.30	4.27	0.974	0.913	0.29	4.14	0.982
U6PS2	0.912	0.81	5.73	0.981	0.912	0.20	2.83	0.995
U7BS1	0.881	0.74	5.21	0.952	0.972	0.83	5.89	0.963
U7BS2	0.915	0.60	4.24	0.971	0.888	0.15	2.13	0.995
U7PS1	0.871	0.24	3.37	0.984	0.950	0.85	6.02	0.960
U7PS2	0.918	0.22	3.11	0.989	0.882	0.70	4.96	0.977
U6RL	0.956	0.15	1.09	0.991	0.950	0.16	1.13	0.986
U7RL	0.991	0.35	1.66	0.978	0.889	0.16	1.16	0.990

*Note*: ICC: Intraclass correlation coefficient TEM and rTEM indicate an absolute and relative technical error of measurement

### Bone thickness

Comparing pre- (T0) and post-treatment (T1) alveolar bone thickness of the maxillary molars, the means changes were found that reduction in alveolar bone thickness in the average buccal surface of the maxillary 1st molar and the buccal and palatal surfaces of the maxillary 2^snd^ molar. In contrast, the palatal surface at S1 and the average bone thickness had significantly increased in the maxillary 1st molar (*p* < 0.005) Table 2.

**Table 2:** Comparison(T0-T1) of maxillary alveolar bone thickness and root length of 1st and 2nd molar teeth

			pre	post	T0-T1	p.v
First Molar	buccal	s1	1.73 ± 0.76	1.64 ± 0.68	0.09 ± 0.32	0.125
		s2	2.07 ± 0.91	2.13 ± 0.95	-0.07 ± 0.44	0.418
		average	1.90 ± 0.76	1.88 ± 0.75	0.01 ± 0.29	0.813
	palatal	s1	1.70 ± 0.42	1.82 ± 0.45	-0.12 ± 0.32	0.045
		s2	2.59 ± 0.89	2.52 ± 0.91	0.07 ± 0.30	0.218
		average	1.76 ± 0.40	2.17 ± 0.62	-0.41 ± 0.40	0.000
Second Molar	buccal	s1	2.47 ± 0.60	2.40 ± 0.53	0.07 ± 0.37	0.277
		s2	3.31 ± 0.67	3.36 ± 0.73	-0.05 ± 0.39	0.461
		average	2.89 ± 0.56	2.88 ± 0.56	0.01 ± 0.32	0.858
	palatal	s1	2.21 ± 0.54	2.18 ± 0.50	0.03 ± 0.35	0.688
		s2	2.72 ± 0.65	2.61 ± 0.71	0.12 ± 0.38	0.104
		average	2.47 ± 0.55	2.39 ± 0.55	0.07 ± 0.31	0.224
OIIRR						
First Molar	right		12.69 ± 1.21	12.24 ± 1.10	0.46 ± 0.47	0.000
	left		12.72 ± 1.42	12.28 ± 1.18	0.45 ± 1.09	0.032
	average		12.71 ± 1.01	12.26 ± 0.91	0.45 ± 0.61	0.000
Second Molar	right		12.61 ± 1.26	12.12 ± 1.23	0.49 ± 0.81	0.002
	left		12.13 ± 2.35	11.99 ± 1.12	0.14 ± 1.99	0.702
	average		12.37 ± 1.37	12.06 ± 0.91	0.32 ± 1.20	0.158

### Root resorption

The mean root length value between T0 and T1 showed that post-treatment root length significantly reduced (OIIRR) in the 1st molar roots (*p* < 0.000) Table 2.

## Discussion

The morphology of the maxillary alveolar cortex plays an essential role in orthodontic treatment, particularly in cases where there is a significant discrepancy between the amount of available space in the dental arches and the volume of teeth. Distal movement of the alveolar cortex should be considered in molars during treatment [22]. Excessive movement can lead to undesirable side effects for the periodontal tissue, including attachment loss, bone loss, gingival recession, and root resorption [23]. 

The primary purpose of the current study was to evaluate the impact of clear aligners on the thickness of buccal and palatal alveolar bone, as well as root length, following horizontal movement of molars in skeletal Class II malocclusion. The anatomical thickness of alveolar bone in molar teeth after distalization by clear aligner therapy was measured using CBCT, which provides distortion-free slice images [24]. These images enhance the possibility of evaluating more tooth surfaces compared to conventional radiography [25]. Clear aligner therapy is widely used by clinicians worldwide as an alternative to conventional fixed orthodontic treatments, particularly among adult patients who prioritize aesthetic considerations. In our study, the patient cohort consisted of adults undergoing a standardized treatment protocol for maxillary sequential molar distalization, specifically targeting a minimum of 2 mm actual molar distalization, in non-extraction cases. The maxillary first molars were successfully displaced distally by an average of 2.23 mm. This finding aligns with previous studies demonstrating the feasibility of posterior teeth distalization to address Class II malocclusion using clear aligner therapy [20, 26]. Simon et al. [27] reported a high precision (88%) in the upper molar’s bodily movement with aligners, with a prescribed mean distalization movement of 2.7 mm. Conversely. Brezniak et al. [28] suggested that bodily movement with aligners is not achievable, even when composite attachments are utilized. While various studies have explored the biomechanical mechanisms of clear aligners and their treatment outcomes, the side effects exerted by these treatments remain insufficiently understood [29, 30]. 

In the present study, the mean change in alveolar bone thickness on the buccal surface of all maxillary molars demonstrated a reduction at S1. This result aligns with findings by Juliana et al. [31], who showed a significant decrease in maxillary buccal alveolar bone thickness after treatment with Damon 3MX brackets without extractions, particularly around the 1st molar at S1 (0.6 ± 0.6). Similarly, our findings revealed a reduction in bone thickness on the palatal surface at S2 of the molars, corroborating a CBCT study by Maspero et al. [32], which indicated a decrease in palatal bone thickness at the same location (0.0 ± 0.4). Another study highlighted a significant reduction in the bone thickness for maxillary anterior teeth in the palatal area at the middle of the root length (0.11 ± 0,58) [23]. This was study confirmed these observations, showing a loss of bone thickness on the palatal surface at S2 of both molars (0.07 ± 0.30, 0.12 ± 0.38).

Regarding the average differences in T0-T1 values for maxillary molars, our results found that alveolar bone reduction was more pronounced on the buccal and palatal surfaces of the maxillary 2nd molars and the buccal surface of the 1st molar. Conversely, there was a significant increase in bone thickness on the palatal surface of the 1st molar (*p* < 0.005). These findings contrast with those of Fayed et al. [33] and Swasty et al. [34] reported a significant increase in buccal and palatal bone thickness in both the anterior and posterior regions of the maxilla. The discrepancy may be attributed to the unique type of tooth movement induced by aligners. Prior to the establishment of sequential distalization techniques, the most accurate achieved by aligners is the buccolingual tipping. This is due the materials of the appliance primarily bending in the buccolingual direction, which is in line with the logical mechanics of tooth movement. As a result, arch expansion and buccal tilting of the maxillary molars may lead to palatal tip drooping, causing resorption of the alveolar bone thickness [35, 36]. 

A previous study found that clear aligners effectively treated simple malocclusions with minimal OIIRR in Class I malocclusion patients [8]. Our study observed significant OIIRR in the 1st molar roots (0.45 ± 0.61, *p* < 0.000). Although our research didn’t directly compare aligners to fixed appliances, recent studies focusing on anterior teeth have found that the clear aligner experienced lower prevalence and less severe root resorption (68.50% and 0.31 ± 0.42 mm, respectively) compared to the fixed appliance (82.50% and 0.62 ± 0.54 mm, respectively) [37]. Additionally, prior research has documented that the average OIIRR during comprehensive treatment with fixed appliances ranges between 1.36 and 1.42 mm [38]. An analysis of periapical radiographs indicated that the average OIIRR for maxillary incisors treated with fixed appliances was 2.26 mm [39]. Conversely, a CBCT study found that patients with fixed appliances experienced an average resorption of 0.59 mm in their maxillary incisors [40]. Regarding clear aligners, 2 mm maxillary incisor OIIRR was reported based on the panoramic, periapical, and cephalometric radiographs [41]. However, when using CBCT imaging, one study reported an average root resorption of 0.51 mm in maxillary incisors [42], while another noted a reduction in root length of 0.13 mm for the same teeth [37].

To our knowledge, only one recent study has evaluated OIIRR in posterior teeth following clear aligner treatment. Our study indicated an average maxillary molar root resorption of 0.45 mm after clear aligner therapy, which aligns with the study of Elfouly et al. [26] who reported that the amount of root resorption observed in all studied molars’ roots (< 1 mm) was not clinically significant. The divergent results regarding OIIRR outcomes may stem from differences in the imaging tools selection, sample size, the magnitude of applied force, and the type of appliance used. Unlike fixed appliances, previous research has suggested that clear aligners are associated with a lower risk of OIIRR, likely due to the gentle and intermittent forces exerted during treatment [43, 44]. Recent progress in three-dimensional imaging and rapid prototyping technologies is revolutionizing the workflow in orthodontic clinical practices. Recent research indicates that the specific type of prototyping technology used, as well as the complexity and features of the components in three-dimensional printers, can significantly affect the precision of orthodontic models created for manufacturing clear aligners [45, 46]. These advancements suggest a critical evaluation of the technology and methods used in order to ensure the highest accuracy in orthodontic treatments.

We must acknowledge certain limitations of this study, including the analyses conducted immediately post-treatment without considering follow-up images. As a result, it is unclear if the observed defects resolve spontaneously over time. Additionally, the assessment of significant changes in alveolar bone thickness was hindered by an unequal gender distribution in our study cohort. Variations in hormonal changes between males and females and across different age groups might affect bone remodeling during orthodontic tooth movement. To better understand the changes in bone morphology due to clear aligner treatment, further clinical studies are recommended. Future research should include a comprehensive evaluation of morphology changes across all types and surfaces of teeth and examine the effects of different malocclusions on bone changes during orthodontic treatment.

## Conclusion

- The buccal alveolar bone thickness of maxillary molars is considered the most affected surface following distalization movement using clear aligners, followed by the palatal surface.
- The palatal alveolar bone thickness of 1st molars indicated increased bone thickness.
- The maxillary first molar roots on both sides were significantly shortened after treatment.

## Data Availability

All data generated or analyzed during this study are included in this published article.

## Abbreviations

OIIRR: Orthodontically induced inflammatory root resorption
CBCT: Cone Beam Computed Tomography
T0: before treatment
T1: after treatment
VDA: Vertical dental axis
DICOM: Digital Imaging and Communication in Medicine
CEJ: Cementoenamel junction
